# MoVrp1, a putative verprolin protein, is required for asexual development and infection in the rice blast fungus *Magnaporthe oryzae*

**DOI:** 10.1038/srep41148

**Published:** 2017-01-24

**Authors:** Lin Huang, Shengpei Zhang, Ziyi Yin, Muxing Liu, Bing Li, Haifeng Zhang, Xiaobo Zheng, Ping Wang, Zhengguang Zhang

**Affiliations:** 1 Department of Plant Pathology, College of Plant Protection, Nanjing Agricultural University, and Key Laboratory of Integrated Management of Crop Diseases and Pests, Ministry of Education, Nanjing 210095, China; 2 College of Forestry and Co-Innovation Center for Sustainable Forestry in Southern China, Nanjing Forestry University, Nanjing, Jiangsu 210037, China; 3 Department of Pediatrics, Louisiana State University Health Sciences Center, New Orleans, Louisiana 70118, USA

## Abstract

Endocytosis is a crucial cellular process in eukaryotic cells which involves clathrin and/or adaptor proteins, lipid kinases, phosphatases and the actin cytoskeleton. Verprolin proteins, such as Vrp1 in *Saccharomyces cerevisiae*, are conserved family proteins that regulate actin binding and endocytosis. Here, we identified and characterized MoVrp1 as the yeast Vrp1 homolog in *Magnaporthe oryzae*. Deletion of the *MoVRP1* gene resulted in defects in vegetative growth, asexual development, and infection of the host plant. The *∆Movrp1* mutants also exhibited decreased extracellular peroxidase and laccase activities and showed defects in colony pigmentation, hyphal surface hydrophobicity, cell wall integrity, autophagy, endocytosis, and secretion of avirulent effector. Our studies provided new evidences that MoVrp1 involved in actin cytoskeleton is important for growth, morphogenesis, cellular trafficking, and fungal pathogenesis.

Membrane trafficking or intracellular transport is an important cellular process by which membrane materials including proteins, lipids, and other macromolecules are shuttled between endomembrane compartments and plasma membrane^1^. Endocytosis mediates the internalization, sorting and degradation of endocytosed molecules, and has a crucial role in recycling of plasma membrane lipids and trafficking proteins, and in uptake or downregulation of cell surface receptors in eukaryotic cell^2^,^3^. The endocytic pathway was visualized first time with the membrane-selective marker dye FM4-64 in yeast *Saccharomyces cerevisiae*^4^. Recently studies showed that endocytosis is conserved from yeast to filamentous fungi, and mammalian cells, which emphasizes the importance of endocytosis for eukaryotic cells^5^. In yeast and plant pathogenic fungi, endocytosis is extensively involved in cell polarity establishment, hyphal growth, and/or virulence^6^,^7^,^8^,^9^,^10^,^11^.

Endocytosis is directly linked to the actin cytoskeleton that consists of actin patches, actin cables and the contractile ring^12^,^13^. There is a clear link between actin patches and sites of endocytosis at the plasmamembrane^14^. Actin patches exhibit rapid movement and facilitate internalization of early endosomes, the primary function of which is the sorting of internalized cargo to different intracellular destinations^15^. In yeast, several actin-binding proteins such as Abp1, Srv2/End14, Rvs161/End6, Rvs167 and Sla2/End4 have been demonstrated to participate in endocytosis, and in resistance of external stresses^16^,^17^. As a key regulator of cortical actin-patch distribution and endocytosis, verprolin (Vrp1/End5) was originally discovered in *S. cerevisiae*^18^. Verprolins were also identified in the genome of other eukaryotic organisms, including nematodes and insects, each of which has a single-copy verprolin gene, while vertebrates have three genes of verprolin-like protein^19^. The human verprolin family consists of the Wiskott-Aldrich syndrome protein (WASP)-interacting protein (WIP), glucocorticoid-regulated gene-product (CR16) and WIP-related (WIRE)^20^,^21^,^22^. Verprolins have important roles in signaling to actin dynamics which were mainly mediated by the WASP proteins, and influence the actin polymerisation machinery independent on the WASP proteins^19^.

In yeast, Vrp1 displays a subcellular distribution polarized towards sites of surface growth and partially co-localized with cortical actin patches^23^,^24^. Vrpl was shown similar function to the type I myosins Myo3 and Myo5, the WASP homolog Las17/Beel and the Arp2/3complex to orchestrate actin organization. Vrp1 is required for cell growth, cytoskeletal organization, endocytosis, cytokinesis and mitochondrial protein distribution and function^24^. Loss of Vrp1 leads to defects in polarization of cortical actin patches, internalization of receptor-bound and fluid-phase endocytic cargo, and inviability at elevated temperatures^18^,^24^,^25^.

Despite these important findings, functions of Vrp1 proteins in filamentous fungi remain unclear. In the rice blast fungus *M. oryzae*, infection is carried out by the conidia which germinates to produce an appressirium^26^,^27^. Endocytosis plays a crucial role in conidial cells to detect certain external signals, and is required for hyphal tip growth^28^. Previously, we characterized two members of the secretory soluble N-ethylmaleimide-sensitive factor attachment protein receptors (SNAREs), MoSec22 and MoVam7 in *M. oryzae*, which are required for membrane trafficking, cellular growth, stress tolerance and pathogenicity^7^,^10^. Our previous studies also showed that MoArk1, as a homolog of Ark1 in *S. cerevisiae*, which showed an actin-like localization pattern by localizing to the hyphal apices strongly affects organization of the cortical actin cytoskeleton. Deletion of *MoARK1* resulted in defects in mycelial growth, conidial production, and pathogenicity, and caused defects in endocytosis and formation of the Spitzenkörper^29^. Very recently, we found that deletion of the Qc-SNARE protein MoSyn8 resulted in reduction and alternation in distribution of F-actin patches. We also found that MoSyn8 regulates the effector secretion in the early stage of infection. The endocytosis mediated by MoSyn8 is required for normal physiology and pathogenesis of *M. oryzae*^30^. In this study, we identified and characterized the yeast Vrp1 homolog MoVrp1 in *M. oryzae*, and showed that MoVrp1 is not only localized to the actin patches but also important for endocytosis, hyphal growth, conidial development, stress response, cell wall integrity, and pathogenicity.

## Results

### Identification and deletion of MoVrp1 in *M. oryzae*

Using the *S. cerevisiae* Vrp1 sequence as the reference to search the *M. oryzae* genome database (http://www.broad.mit.edu/annotation/genome/magnaporthe_grisea/Home.html), we identified the MGG_11243.6 genetic loci encoding the Vrp1 homolog MoVrp1. MoVrp1 contains 466 amino acids sharing 50 and 69% amino acid sequence identity with yeast Vrp1 and *Neurospora crassa* Vrp1, respectively. Phylogenetic analysis of fungal Vrp1 proteins showed that the Vrp1-like proteins in filamentous fungi have diverged from those of unicellular yeasts, with MoVrp1 being the most similar to *N. crassa* Vrp1 (XP_963859) (Fig. S1A).

To investigate the roles of MoVrp1, gene deletion mutants were generated by replacing the MoVrp1 coding region with the hygromycin phosphotransferase resistance (*HPH*) gene. Candidate mutants were confirmed by Southern blot and semi-quantitative RT-PCR analysis, and two *MoVRP1* gene deletion mutants, *∆Movrp1*#54 and *∆Movrp1*#105, were obtained (Fig. S1B and S1C). Southern blot analysis showed that the *Movrp1* gene is present as a single copy in the *M. oryzae* genome (Fig. S1C).

### MoVrp1 is important for vegetative growth and colony pigmentation

**Figure 1:**
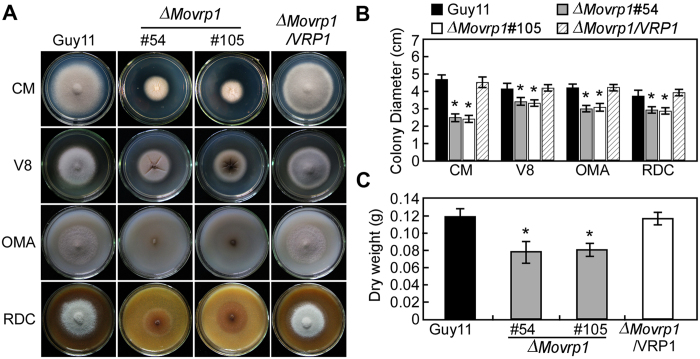
MoVrp1 is essential for normal mycelial growth. (**A**) Colony morphology of the wildtype Guy11, ∆*Movrp1*#54, ∆*Movrp1*#105 and complemented strain grown on CM, V8, OMA and SDC media plates for 7 days. (**B**) Colony diameter of Guy11, ∆*Movrp1*#54, ∆*Movrp1*#105 and complemented strain grown on CM, V8, OMA and SDC media plates for 7 days. (**C**) The dry weight of mycelia from the wildtype Guy11, ∆*Movrp1*#54, ∆*Movrp1*#105 and complemented strain cultured at 150 rpm for 2 days at 28 °C. Each experiment was repeated three times with similar results. Error bars represent standard deviation from the mean, and *t* test analysis is shown with *p < 0.01 *versus* the wildtype Guy11.

**Figure 2:**
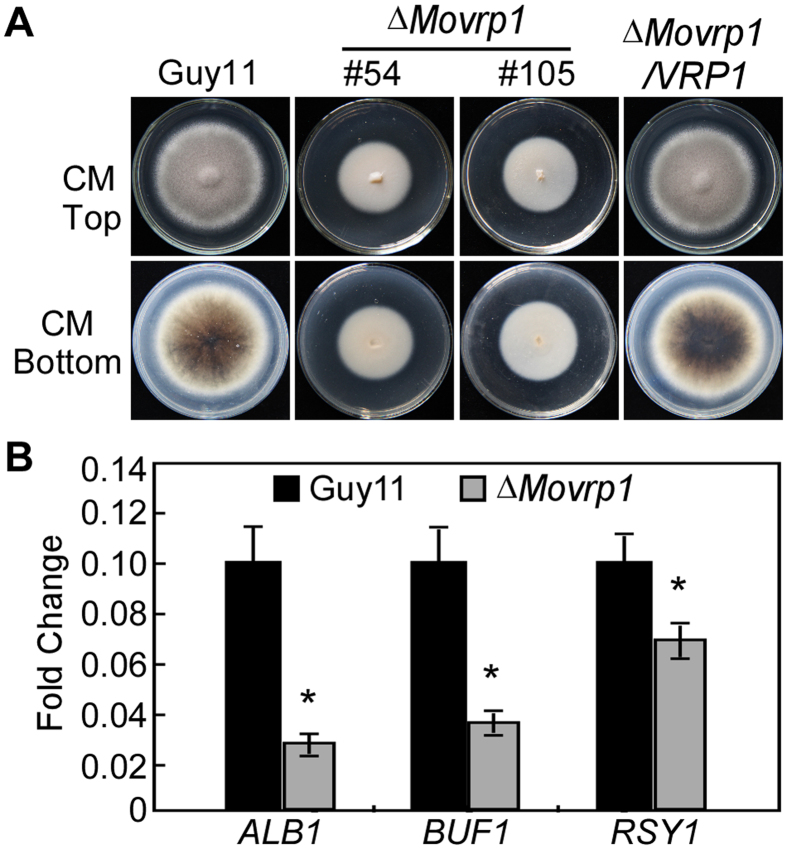
MoVrp1 is involved in colony pigmentation. (**A**) Colony morphology of the wildtype Guy11, ∆*Movrp1*#54, ∆*Movrp1*#105 and complemented strain was observed on CM agar plates in the dark for 7 days at 28 °C. (**B**) Expression of three pigmentation associated genes in the wildtype Guy11 and ∆*Movrp1* mutant. The experiments were repeated three times with triple replications. Error bars represent standard deviation from the mean, and *t* test analysis is shown with *p < 0.01 *versus* the wildtype Guy11.

To address the role of MoVrp1 in vegetative growth, the wildtype Guy11, *∆Movrp1* mutants and complemented strain *∆Movrp1*/*VRP1* were inoculated onto CM, V8, OMA, and SDC media plates, respectively. Compared to the wildtype Guy11 and complemented strain, the *∆Movrp1* mutants showed reduced growth and lacked aerial hyphae on various media (Fig. 1A and B). Similar to the results on CM agar plates, the *∆Movrp1* mutants also showed shorter hyphae and the dry weight decreased to 70% of the wildtype Guy11 in liquid CM (Fig. 1C). Additionally, the colonies of *∆Movrp1* showed absolutely no pigmentation (Fig. 2A), and the expression of three pigmentation biosynthesis-related genes was decreased significantly in the *∆Movrp1* mutant (Fig. 2B). These results indicated that MoVrp1 plays a crucial role in vegetative growth and colony pigmentation in *M. oryzae*.

### MoVrp1 is indispensable for asexual development

**Figure 3:**
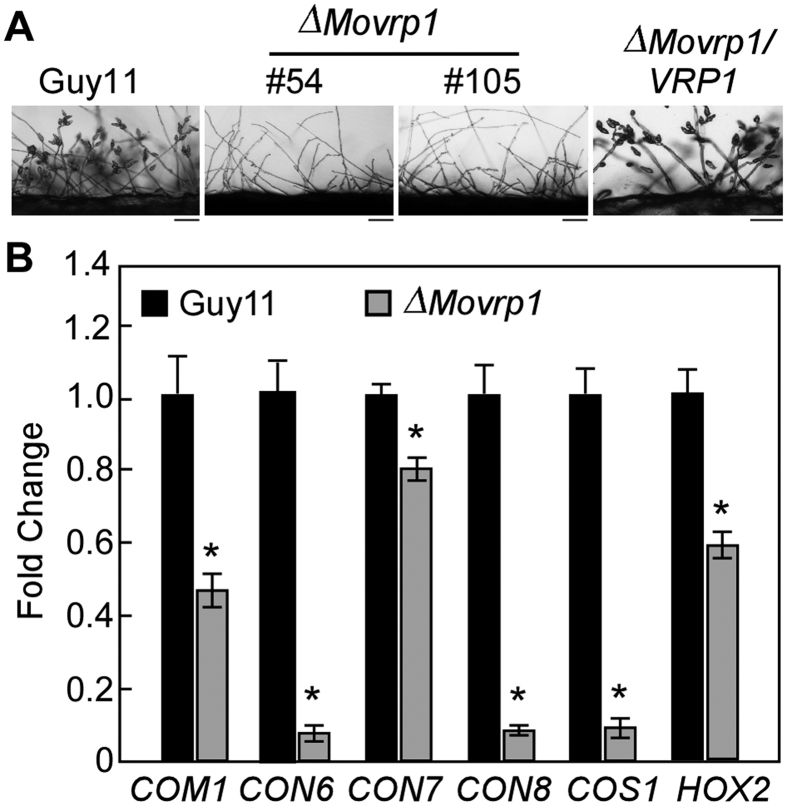
MoVrp1 is required for conidiation. (**A**) Seven-day-old hyphal blocks were placed on glass slides for 24 hours to promote conidiation. Bars = 50 μm. (**B**) Expression of six conidiation associated genes in the wildtype Guy11 and ∆*Movrp1* mutant. The experiments were repeated three times with triple replications. Error bars represent standard deviation from the mean, and *t* test analysis is shown with *p < 0.01 *versus* the wildtype Guy11.

Asexual spores are important for the disease cycle of *M. oryzae*^27^. No conidia were observed in *∆Movrp1* mutants, and their ability to produce conidia was completely abolished. In contrast, the wildtype Guy11 and complemented strain produced a large number of conidia (Fig. 3A). Furthermore, quantitative RT-PCR (qRT-PCR) analysis showed that the expression levels of five conidiation-related genes was significantly downregulated in *∆Movrp1* mutant (Fig. 3B). These results suggested that MoVrp1 is indispensable for conidial production by modulating the expression of several conidiation-related genes in *M. oryzae*.

### MoVrp1 is required for virulence and is important for appressorium-like structure formation

**Figure 4:**
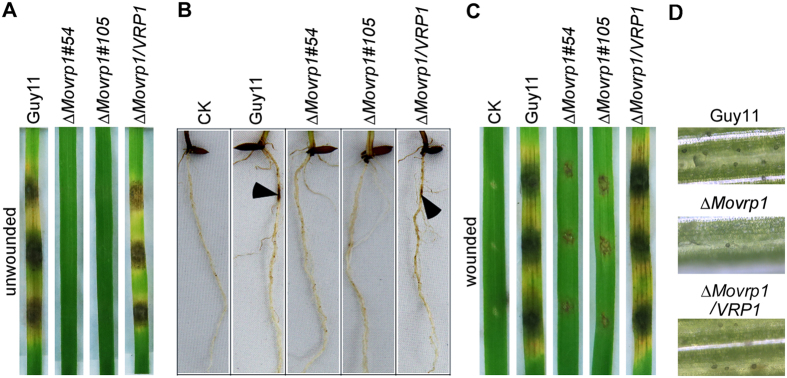
The ∆*Movrp1* mutants were nonpathogenic on detached rice leaves. (**A** and **C**) unwound and wounded rice (CO-39) leaves were inoculated with mycelial plugs of the wildtype Guy11, ∆*Movrp1*#54, ∆*Movrp1*#105 and the complemented strain, and photographed at 7 day post-inoculation, respectively. (**B**) Rice roots were inoculated with the wildtype Guy11, ∆*Movrp1*#54, ∆*Movrp1*#105 and the complemented strain, respectively. (**D**) Appressorium-like structures were formated on rice leaves. Mycelial mats were inoculated on rice leaves and photographed at 48 h post-inoculation. The experiments were repeated three times with the similar results.

Normally, *M. oryzae* invades rice cells by appressoria, which develop from conidia^27^. However, *M. oryzae* also forms appressorium-like structures at the hyphal tips on plant surfaces to facilitate breaching the plant cuticle and causing disease^31^. Since *∆Movrp1* mutants were unable to produce spores, mycelial mats of the wildtype Guy11, *∆Movrp1* and complemented strain were inoculated onto detached rice and barley leaves. The results showed that the *∆Movrp1* mutants caused no lesions on the unwounded rice and barley leaves, while the wildtype Guy11 and complemented strain caused typical rice blast lesions (Fig. 4A and Fig. S2A). We further tested the infection on rice roots and observed the same results as on rice leaves (Fig. 4B). To clarify whether MoVrp1 was involved in infectious growth, the indicated strains were inoculated onto the wounded rice and barley leaves, where small and limited lesions were observed from the *∆Movrp1* mutants, compared to typical lesions of the wildtype Guy11 and complemented strain (Fig. 4C and Fig. S2B). We also examined appressorial formation of the hyphal tip on intact rice leaves. Numerous appressorium-like structures were observed in the wildtype Guy11 and complemented strain, while we rarely observed appressorium-like structures in the *∆Movrp1* mutants (Fig. 4D). We concluded that MoVrp1 plays a crucial role in appressorium-like structure formation and pathogenicity in the rice blast fungus.

### MoVrp1 plays a role in responses to various stresses

To test the role of MoVrp1 in response to stresses, the wildtype Guy11, *∆Movrp1* mutants, and complemented strain were inoculated on CM plates where they were subjected to ion stress, osmotic stress, temperature stress, and cell wall stressors, respectively. As indicated in Table 1, the *∆Movrp1* mutants showed higher inhibition rate than the wildtype Guy11 and complemented strain when they were exposed to NaCl, KCl and sorbitol. When the *∆Movrp1* mutants were cultured at different temperatures, compared to the mycelia growth at 28 °C, the growth inhibition rate of *∆Movrp1* mutants was significantly less than the wild type and complemented strain at 20, 25, 30 and 33 °C (Table 1). We also tested the cell wall stressor sensitivity of the mutants when inoculated on CM plates containing various concentrations of sodium dodecyl sulfate (SDS), Calcofluor white (CFW) and Congo red (CR), respectively. The results showed that the inhibition rate was significantly decreased in the *∆Movrp1* mutants when treated with different cell wall stressors (Table 2). These results suggested that MoVrp1 plays an important role in response to environmental stresses in *M. oryzae*.

### MoVrp1 plays a crucial role in cell wall integrity

**Figure 5:**
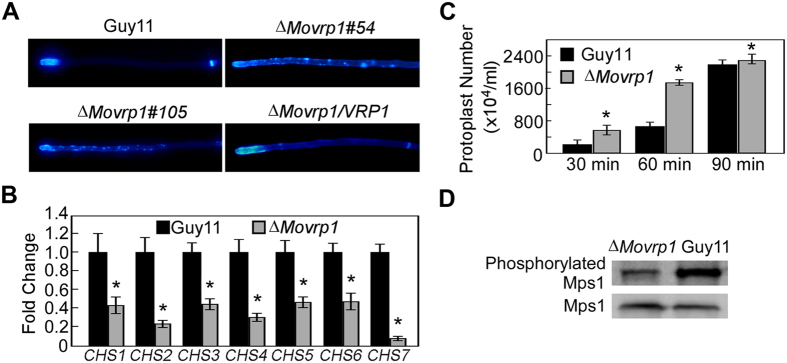
Deletion of *MoVRP1* altered the distribution of chitin. (**A**) Disruption of *MoVRP1* altered the chitin distribution on the cell wall. Hyphae of the wildtype Gyu11, ∆*Movrp1*#54, ∆*Movrp1*#105 and the complemented strain were stained by 10 μg/mL CFW for 2 min and then photographed. (**B**) Expression levels of seven *CHS* genes in the wildtype Guy11 and ∆*Movrp1* mutant. (**C**) Protoplasts of the wildtype Guy11 and ∆*Movrp1* mutant were counted when the mycelia were treated 30, 60 and 90 min with the solution of cell wall chitinase. (**D**) Western blot analysis of phosphorylation level of Mps1. The activation was detected with an anti-MAPK antibody and an anti-Actin was used as a positive control. All experiments were repeated three times with triple replications. Error bars represent standard deviation from the mean, and *t* test analysis is shown with *p < 0.01 *versus* the wildtype Guy11.

Fungal cell wall is important for maintaining cell morphology and adaptation to the extracellular environment^32^. As chitin is a major component of the filamentous fungal cell wall, the normal synthesis and distribution of chitin is the key to maintaining polar hyphal tip growth and hyphal morphology^33^,^34^. Chitin distribution was examined by CFW staining and showed that chitin of the wildtype Guy11 and complemented strain accumulated mainly in hyphae tips. However, in the *ΔMovrp1* mutants, the chitin was not only restricted to the growing apices, but also distributed in the lateral walls along hyphal axes (Fig. 5A). Further qRT-PCR analysis showed that the expression levels of seven chitin synthesis-related genes was significantly decreased in the *∆Movrp1* mutants (Fig. 5B), indicating that *∆Movrp1* mutants have defects in chitin synthesis and distribution. Protoplast release assays showed that the*∆Movrp1* mutant released protoplasts more quickly than the wildtype Guy11 (Fig. 5C). These results indicated that the *∆Movrp1* mutants were defective in cell wall integrity. In *M. oryzae*, the Mps1 mitogen-activated protein kinase (MAPK) pathway is essential for cell wall integrity^31^,^35^. To find out whether the cell wall defects were related to the Mps1 MAPK pathway, we detected the phosphorylation activity of Mps1 based on western blot analysis. The results showed that the 44-kDa Mps1 signals were significantly decreased in *∆Movrp1* compared to the wildtype Guy11 (Fig. 5D). We concluded that cross talk occurs between MoVrp1 and the Mps1 MAPK pathway and that MoVrp1 modulates Mps1 phosphorylation activity, and therefore regulates the cell wall integrity of *M. oryzae*.

### MoVrp1 is essential for surface hydrophobicity

**Figure 6:**
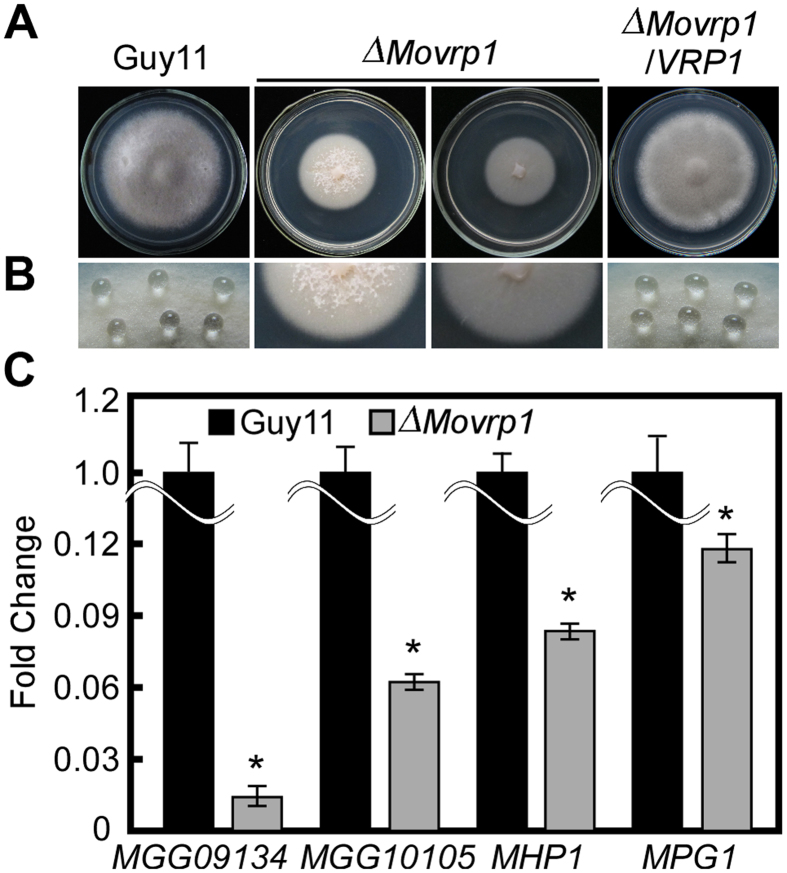
MoVrp1 is required for surface hydrophobicity. (**A**) Colony morphology of Guy11, ∆*Movrp1*#54, ∆*Movrp1*#105 and the complemented strain cultured on CM media plate. Drops of ddH_2_O (upper row) and detergent solutions (lower row) containing 0.02% SDS and 5 mM EDTA were used to determine the surface hydrophobicity. Liquid drops was add to the surface of 7 d hypha for 5 min. (**B**) Expression analysis of four hydrophobicity-related genes *MGG_09134, MGG_10105, MoMPG1* and *MoMHP1* in the wildtype Guy11 and the ∆*Movrp1* mutant. The experiment was repeated three times with triple replications. Error bars represent standard deviation from the mean, and *t* test analysis is shown with *p < 0.01 *versus* the wildtype Guy11.

Surface hydrophobicity is important for normal mycelia development and infection in *M. oryzae*^36^,^37^. To determine whether MoVrp1 was involved in mycelia surface hydrophobicity, water and detergent solutions were dropped onto the surfaces of the wildtype Guy11 and *∆Movrp1* strains. Droplets of water and detergent solutions immediately soaked into the mutant cultures, but not into the wildtype Guy11 and complemented strain (Fig. 6A). We further examined the expression levels of hydrophobicity-related genes including *MoMPG1, MoMHP1, MGG_09134*, and *MGG_10105*. Compared to the wildtype, the expression levels of these genes were significantly decreased in the *∆Movrp1* mutant (Fig. 6B). This result indicated that MoVrp1 plays a crucial role in regulating the surface hydrophobicity in *M. oryzae*.

### MoVrp1 is involved in endocytosis and secretion of the effector Avr-Pia

**Figure 7:**
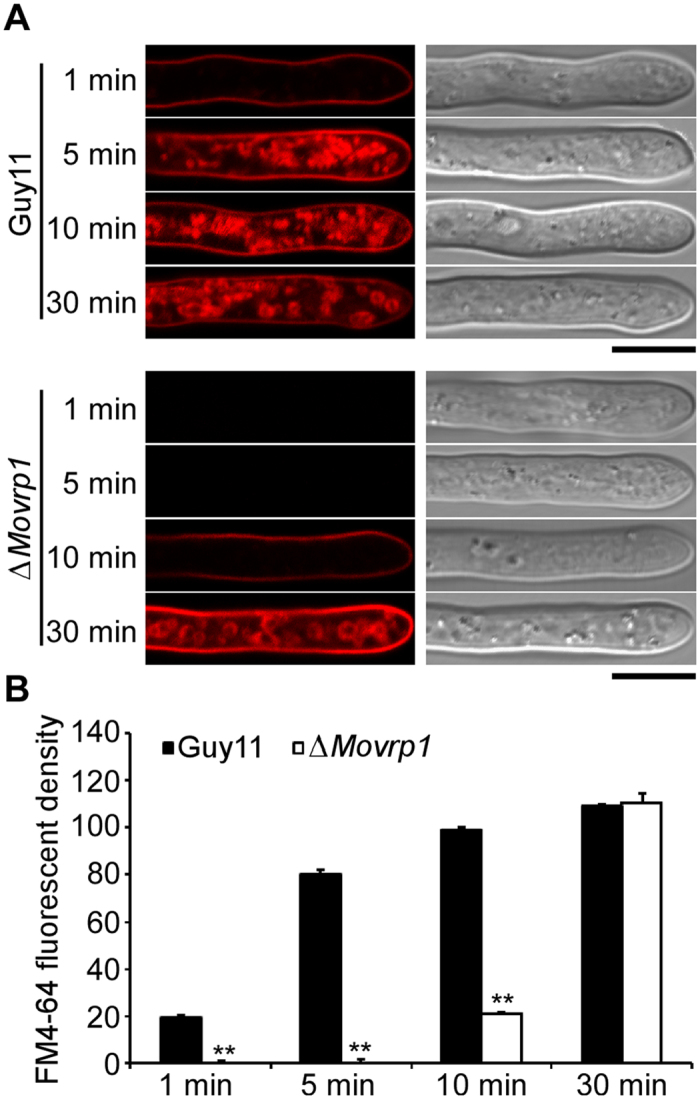
MoVrp1 is involved in endocytosis. (**A**) 5 × 5 mm hyphal plugs of wildtype Guy11, and ∆*Movrp1* mutant was inoculated on CM lipid medium for 2 days, then the mycelia were collected and stained by the FM4-64 by time course. Bars = 10 μm. (**B**) Statistical analysis of FM4-64 fluorescent density of the indicated strains. The experiment was repeated three times with triple replications. Error bars represent standard deviation from the mean, and *t* test analysis is shown with *p < 0.01 *versus* the wildtype Guy11.

To assess the role of MoVrp1 in endocytosis, the fluorescent dye FM4-64 was used to visualize the internalization of vacuolar membranes. In wildtype hyphal cells, FM4-64 was internalized within 5 min after staining and resulting in large number of ring-like structures corresponding to endosomes and vesicles. These structures were also observed in the complemented strain (Fig. 7A and B). In contrast, no definitive staining pattern was observed in the *∆Movrp1* mutant within 10 min, and similar results to those in wildtype Guy11 were observed in the mutants until 30 min (Fig. 7A and B). These results indicated that endocytosis was delayed in the *∆Movrp1* mutants.

**Figure 8:**
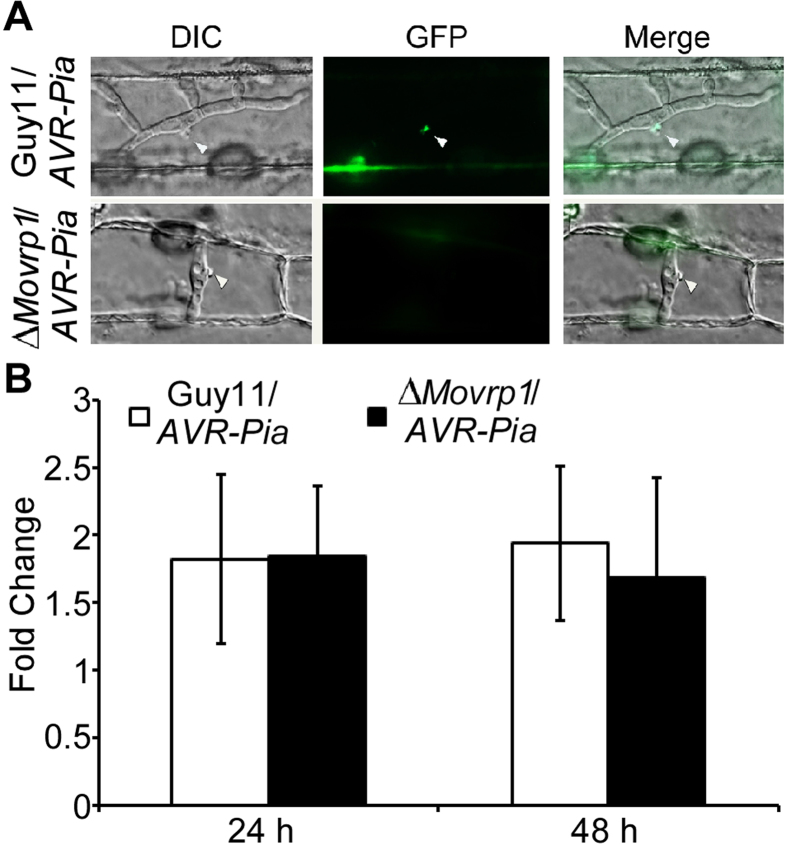
MoVrp1 is required for secretion of effector Avr-Pia. (**A**) The wildtype Guy11 and ∆*Movrp1* mutant overexpressed *AVR-Pia::GFP* were inoculated on epidermal barley cells at 48 hpi and observed fluorescence at BICs. BICs are indicated by arrows. (**B**) The expression level of *AVR-Pia* in response to infection by the mycelium mats of the wildtype Guy11/*AVR-Pia* and the ∆*Movrp1*/*AVR-Pia* at 24 and 48 hours post inoculation (hpi) of the barley leaves. Error bars represent standard deviation from the mean. The experiments were repeated three times with triple replications.

Biotrophic interfacial complexes (BICs) are plant-derivedmembrane-rich structures associated with the accumulation of effectors secreted by *M. oryzae*, which were translocated across the extra-invasive hyphal membrane (EIHM) into the cytoplasm of living host cells^38^,^39^. To determine whether MoVrp1 is involved in the secretion of effectors, *AVR-Pia::GFP* was transformed into the wildtype Guy11 and the Δ*Movrp1* mutant respectively. Both strains were inoculated on barley leaves for 27 h. Compared to numerous primary hyphae were observed in the wildtype Guy11, these structures were rarely observed in Δ*Movrp1* mutant. Microscopy observation of the secreted Avr-Pia::GFP protein also showed strong fluorescence that outlined the primary hyphae and BICs involved in the facilitation of biotrophic invasion in the wildtype Guy11, however, no fluorescence was observed in the Δ*Movrp1* mutant (Fig. 8A). Moreover, there was no significant difference in the expression of *Avr-Pia* between the wildtype Guy11 and Δ*Movrp1* mutant (Fig. 8B). These results suggested that MoVrp1 is involved in the secretion of Avr-Pia but is not required for its expression *in* *planta*.

### MoVrp1 is related to extracellular laccase and peroxidase activities

**Figure 9:**
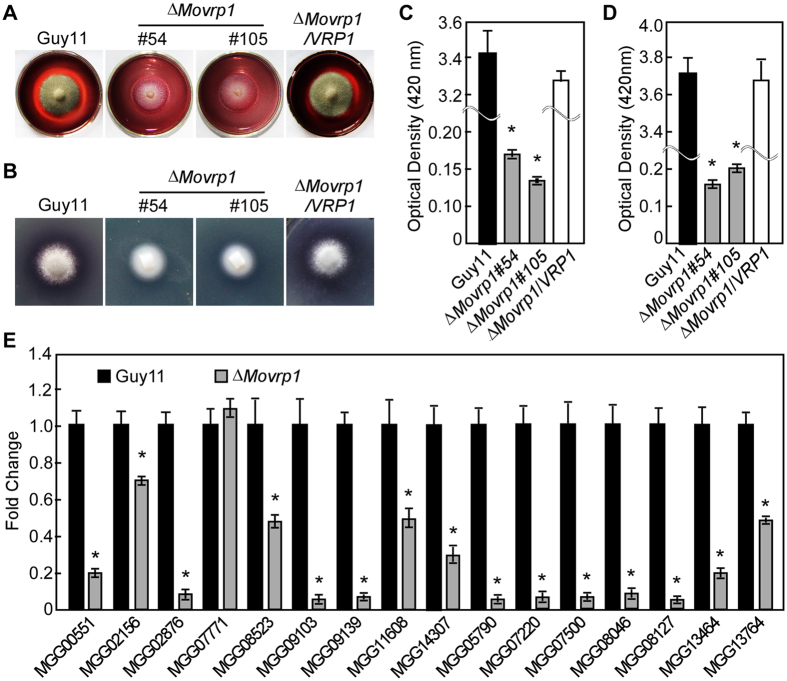
MoVrp1 is involved in extracellular laccase and peroxidase activities. (**A**) The discoloration of CR was tested on CM plates. Strains were inoculated on CM plates containing 200 μg/ml of Congo red and discoloration was observed on day 7 at 28 °C. (**B**) Laccase activities of the wildtype Guy11, ∆*Movrp1*#54, ∆*Movrp1*#105 and complemented strain were tested on CM plates containing 0.2 mM ABTs and photographed after 3 days incubate at 28 °C. (**C**) Extracellular peroxidase activities measured by ABTs oxidizing test under H_2_O_2_ supplemented condition. (**D**) Laccase activities measured by ABTs oxidizing test without H_2_O_2_. (**E**) Expression levels of 16 laccase- or peroxidase-encoding genes in the wildtype Guy11 and the ∆*Movrp1* mutant. All experiments were repeated three times with triple replications. Error bars represent standard deviation from the mean, and *t* test analysis is shown with *p < 0.01 *versus* the wildtype Guy11.

Extracellular peroxidase and laccase are required for the pathogenicity of certain fungi^7^,^34^,^40^,^41^. CR and 2’-azino-di-3- ethylbenzathiazoline-6-sulfonate (ABTs) are used as indicators for the presence of extracellular laccase and peroxidase, respectively^42^. When the *∆Movrp1* mutants were inoculated onto CR-containing medium, the CR degradation halos of the *∆Movrp1* mutants were not as apparent as those of the wildtype Guy11 and complemented strain (Fig. 9A), indicating a deficiency in the CR-degrading activity in the *∆Movrp1* mutants. Extracellular laccase activities of the wildtype Guy11 and *∆Movrp1* mutants were also tested on CM agar plates and culture filtrates with ABTs. No oxidized dark purple stain was observed around the colonies of the *∆Movrp1* mutants on CM agar plates, but obvious dark purple stains occurred around the wildtype Guy11 and complemented strain (Fig. 9B). Further colorimetric assays for filtrates indicated very low peroxidase and laccase activities in the *∆Movrp1* mutants (Fig. 9C and D). In addition, qRT-PCR assay also revealed that the expression levels of 15 laccase- or peroxidase-encoding genes were significantly downregulated in *∆Movrp1* than the wildtype Guy11 (Fig. 9E). These data suggested that MoVrp1 was involved in the regulation of the extracellular peroxidase and laccase activities in *M. oryzae*.

### MoVrp1 is involved in autophagy

**Figure 10:**
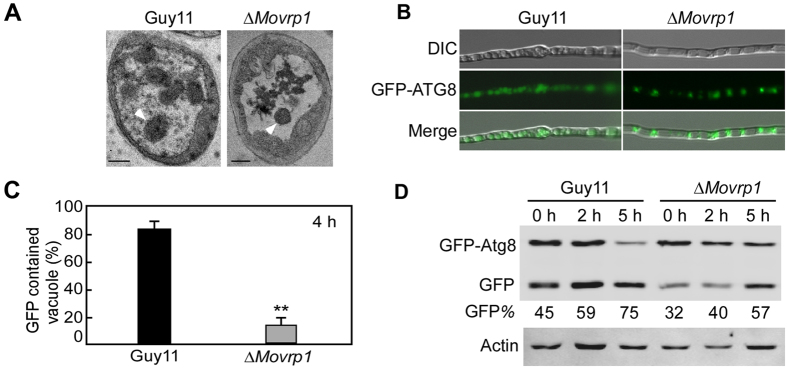
MoVrp1 is involved in autophagy. (**A**) Organelles and autophagic bodies were observed in vacuoles under the starvation condition. Arrowheads represent the autophagic bodies in vacuoles. Bars = 1 μm. (**B**) Guy11 and ∆*Movrp1* mutant expressing GFP-Atg8 were grown in liquid CM medium at 28 °C for 10 h, and shifted to liquid MM-N medium with 2 mM PMSF for 4 h. Hyphae were examined by DIC or epifluorescence microscopy. (**C**) Statistical analysis of the vacuoles contained GFP in hyphae. The experiments were repeated three times with triple replications. Error bars represent standard deviation from the mean, and *t* test analysis is shown with *p < 0.01 *versus* the wildtype Guy11. (**D**) GFP-Atg8 proteolysis assays of Guy11 and ∆*Movrp1* mutant. Mycelia were cultured at 28 °C for 10 h in liquid CM medium and shaken at 150 rpm. Autophagy was induced after 8 h of nitrogen starvation. Mycelia were harvested and mycelia extracts were analyzed by western blot using anti-GFP.

Verprolins have important roles in signaling to actin dynamics^19^, and actin is necessary for starvation-mediated autophagy^43^. To explore whether MoVrp1 plays a role in the autophagy, the vacuoles of hyphal cells were observed after starvation induction under transmission electron microscopy. After culturing in liquid MM-N medium with 2 mM PMSF for 4 h, few autophagic bodies in the vacuole of the *∆Movrp1* mutant were observed. However, numerous autophagic bodies were observed in the wildtype Guy11 (Fig. 10A). The autophagic process can be tracing-observed by monitoring the vacuolar delivery and breakdown of GFP-Atg8^44^,^45^. When induced under nitrogen starvation GFP-Atg8 accumulated in vacuoles of the wildtype Guy11. However, GFP signals remained in the punctuate structures in the *∆Movrp1* mutants (Fig. 10B). Statistical analysis of the vacuoles containing GFP also confirmed this observation (Fig. 10C). To further explain this result, we performed GFP-Atg8 proteolysis assays. Under normal conditions (CM medium), the GFP degradation ratio was 45% in the wildtype Guy11 compared to 32% in the mutants (Fig. 10D). Under induction conditions (MM-N), the GFP degradation ratio was 59% in wildtype Guy11 compared to 40% in the mutant after a 2-h induction, and increased to 75% in wildtype Guy11 and 57% in the mutant after a 5-h induction. However, the GFP degradation in the mutant was significantly lower than that in the wildtype Guy11 at each time point (Fig. 10D). These results indicated that the *∆Movrp1* mutant was defective in autophagy.

### MoVrp1 co-localizes with actin and plays a crucial in regulating the proper localization of actin

**Figure 11:**
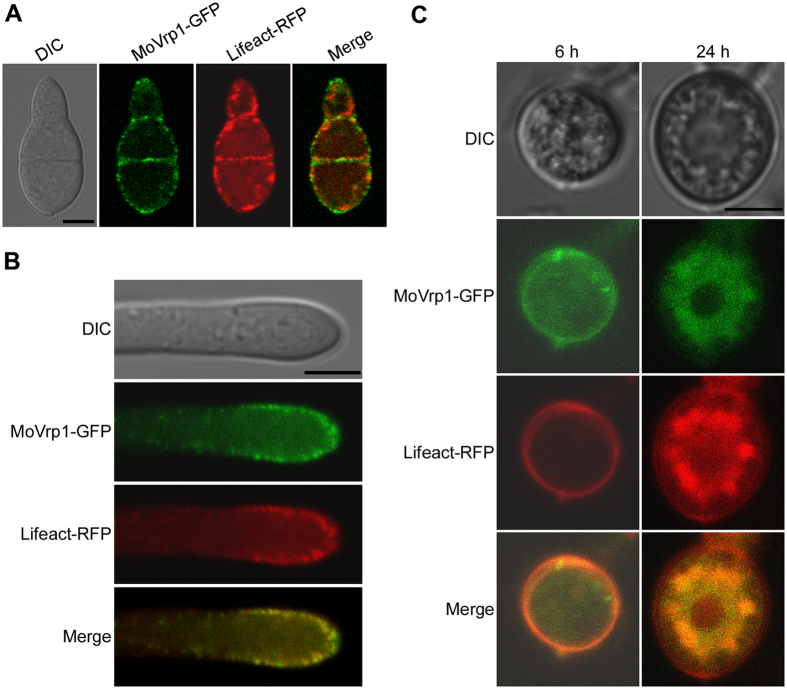
MoVrp1-GFP expression and localization. (**A** and **B**) MoVrp1-GFP and Lifeact-RFP expression and localization in conidium and hyphae, respectively. (**C**) MoVrp1-GFP and Lifeact-RFP expression and localization in appressoria at 6 and 24 hpi, respectively. Bars = 10 μm.

To test the expression and localization pattern of the MoVrp1 protein in *M. oryzae*, a MoVrp1-GFP fusion construct was generated and transformed into the *∆Movrp1* mutant. The conidia, hyphae, and appressoria of the resulting transformants were observed under a fluorescence microscope. Strong GFP signals were mainly observed in the punctate structures, similar to those of actin patches (Fig. 11A). MoVrp1-GFP was distributed in peripherial regions of the conidia, hyphae, and developing appressoria, however, in mature appressoria, MoVrp1-GFP was mainly localized in globular structures in appressorium pore area (Fig. 11A–C). We further transformed a Lifeact-RFP (red fluorescent protein) construct into MoVrp1-GFP strain, and observed Lifeact-RFP co-localization with MoVrp1-GFP in various developmental stages of *M. orzyae* (Fig. 11A–C), suggesting that MoVrp1-GFP was localized to actin in the rice blast fungus.

**Figure 12:**
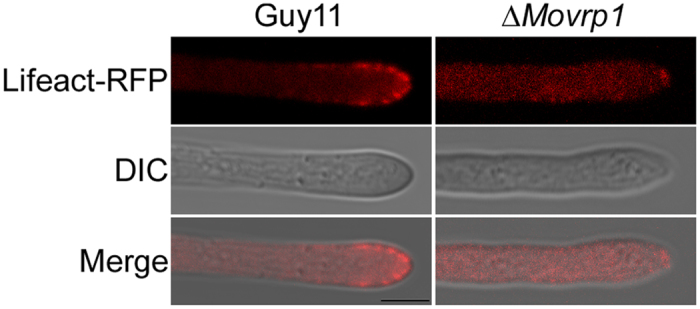
MoVrp1 regulates the proper localization pattern of Lifeact. Lifeact-RFP construct was transformed into wildtype Guy11 and ∆*Movrp1* mutant, respectively. Hyphae from the resulting transformants were observed under an epifluorescence microscopy. Bars = 10 μm.

To explore whether deletion of MoVrp1 affects the localization pattern of Lifeactin, the Lifeact-RFP construct was transferred into the wildtype Guy11 and the *∆Movrp1* mutant. We found that the RFP signals were distributed in the cytosol of the mutant, in contrast to the normal actin patch localization pattern in wildtype Guy11 (Fig. 12), indicating that MoVrp1 plays a crucial in regulating the proper localization of actin in *M. oryzae*.

## Discussion

Endocytosis is a vesicular transport pathway in eukaryotic cells that internalizes extracellular fluid and particles, as well as plasmamembrane molecules^46^. A role of endocytosis in hyphal growth and development, and in fungal pathogenicity in particular, is still subject to debate^47^. However, recent studies showed that a t-SNARE protein, Yup1, is required for endocytosis and pathogenicity in *Ustilago maydis*^8^,^48^. Our previous studies showed that the pathogenicity of the *∆Movam7, ∆Mosec22, ∆M*o*syn8, ∆Moark1*, and *∆Modnm1* mutants, in which endocytosis were delayed or inhibited, and pathogenicity decreased significantly^7^,^10^,^29^,^30^,^45^. In this study, we characterized a yeast Vrp1 homolog protein, MoVrp1 in *M. oryzae*. MoVrp1 is required for the endocytosis and plays important roles in the localization of actin patches, hyphal growth, conidial development, stress response, autophagy and pathogenicity.

Leaf infection by *M. oryzae* initiates from a conidium that germinates and produces the germ tube that further develops into an appressorium which ruptures the rice cuticle and allows a penetration peg entry into the epidermal cells^27^,^39^. *M. oryzae* then specializes invasive hyphae to invade rice tissue, which successively occupy living rice cells and colonize tissue extensively before the appearance of disease symptoms^39^. Internalization of endocytic markers were visualized in conidia cells of *M. oryzae*^6^. In *U. maydis*, an early endosomal t-SNARE protein Yup1 is involved in endocytic recycling, and is required for spore formation and germination, pheromone perception, and cell-cell fusion in the initial steps of pathogenic development^8^,^9^. The *yup1* mutants were defective in uptake of components into early endosomes show heavily altered morphology^48^, and are completely nonpathogenic^8^. These results suggest that there maybe is a link between endocytosis and fungal development and infection of plants. Constant delivery of vesicles and endocytic internalization is required for polarization formation and hyphal growth. As an integral part of the cytoskeleton, actin involves in cell growth, intracellular motility, and cytokinesis in eukaryotic cells^6^,^8^,^11^. Yeast Vrp1 worked in concert with the Arp2/3complex to orchestrate actin organization, as well as endocytosis of the alpha mating factor receptor^18^,^24^,^25^. In this study, MoVrp1 was important for the localization pattern of Life-actin in *M. oryzae. ∆Movrp1* mutants are defective in endocytosis, and deletion of *MoVRP1* altered hyphae growth, conidiation, appressorium-like structure development and pathogenicity in *M. oryzae*. These results indicate that MoVrp1 is required for endocytosis, which regulates the distribution of actin patches, and involves in hyphal polarized growth, invasive structure development and infection of plant in *M. oryzae*.

In yeast cells, *∆vrp1* mutants showed the defects in cytokinesis and grow under elevated temperature^23^. However, deletion of *MoVRP1* resulted in increased resistance to environmental temperatures when compared with the wildtype Guy11, suggesting that MoVrp1 may share distinct regulatory mechanisms against environmental stresses with Vrp1 in yeast. Because the cell wall provides the skeletal support to fungal cells and mediates the interaction between hyphal cells and the surrounding environment, the cell wall integrity and surface hydrophobicity is essential for cell survival in hostile environment and host invasion in pathogenic fungi^41^,^49^,^50^. The *∆Movrp1* mutants altered the resistance to the cell wall stressors SDS, CFW and CR. The chitin was distributed in the lateral walls along hyphal axes in the *∆Movrp1* mutants, but not restricted to the growing apices, as observed in the wildtype Guy11. The transcript levels of seven chitin synthase (CHS) genes were also significantly reduced in the *∆Movrp1* mutant^51^. As one of the key protein kinases of the MAPK cascade which regulates cell wall integrity, the phosphorylation level of MoMps1 is important for cell wall integrity and pathogenicity^31^. Deletion of MoVrp1 significantly attenuated the phosphorylation level of MoMps1. Taken together, these results indicated that MoVrp1 plays a role in cell wall integrity regulation by affecting chitin distribution and the phosphorylation of MoMps1.

Disruption of *MoVRP1* resulted in the defects in surface hydrophobicity and significantly inhibited the expression levels of four hydrophobicity-related genes including *MPG1* and *MHP1*. In *M. oryzae, MPG1* and *MHP1* facilitate fungal spore adhesion, and to direct the action of the enzyme cutinase 2, resulting in penetration of the plant host^52^. Knockout of *MPG1* and *MHP1* results in the impaired appressorium development and reduced infectivity^53^,^54^. Similarly, the *∆Movrp1* mutants showed the defects in appressorium-like structure formation and plant infection. Additionally, hydrophobins can reduce the surface tension of an aqueous growth medium to facilitate production of aerial hyphae and spores^55^. Compared with the wildtype Guy11, the *∆Movrp1* mutant showed thin aerial hyphae and defects in conidiation. qRT-PCR analysis also showed that five conidial development-related genes were downregulated significantly in the *∆Movrp1* mutant. Consistent with these findings, it is indicated the MoVrp1 is required for the surface hydrophobicity, which maybe mediate the regulation of the aerial hyphae growth, conidiation, appressorium-like structure development and pathogenicity in *M. oryzae*.

Secreted peroxidases are important components to help pathogens detoxify host-derived ROS during plant-pathognic fungi interactions, and have key roles in the infection of *M. oryzae*^56^. Laccases are thought to be involved in phytoalexins degradation, melanization synthesis, and contribute to fungal pathogenicity in *M. oryzae* and *Colletotrichum orbiculare*^7^,^34^,^41^,^57^. CR and ABTs were used as substrates to evaluate the activities of extracellular peroxidase and laccase, and the results showed that the activities of extracellular peroxidase and laccase were significantly reduced in the *∆Movrp1* mutants, in contrast to the wildtype Guy11. Moreover, qRT-PCR assay revealed that the expression of 15 laccase- or peroxidase-encoding genes was significantly down-regulated in the *∆Movrp1* mutant. Although the more details about the MoVrp1 regulates the extracellular peroxidase and laccase activity is not very clear yet, our results indicates that MoVrp1 is involved in regulation of extracellular peroxidase and laccase activities in *M. oryzae*. In addition, during the interaction between plant and filamentous fungi, effectors are secreted into plant cells to modulate host defense. In *M. oryzae*, cytoplasmic effector proteins including Pwl2, Avr-Pita, Bas1 and Avr-Piz-t are secreted and accumulate in BICs during biotrophic invasion of rice^39^,^58^,^59^. Previous studies showed that the fungal exocyst components Exo70, Sec5, t-SNARE Sso1, and Syn8 are essential for efficient secretion of cytoplasmic effectors in *M. oryzae*^30^,^39^. Here no fluorescence of the effector Avr-Pia::GFP was observed in the BICs of the *∆Movrp1* mutant, in contrast to the strong fluorescence signal in the wildtype. However, there was no significant difference in the expression level of *AVR-Pia* between the *∆Movrp1* mutant and wildtype Guy11. These results indicated that MoVrp1 might be involved in the secretion of cytoplasmic effectors in *M. oryzae*. Consistent with the reduced virulence of the *∆Movrp1* mutants, we suspect that the defects of pathogenicity of the *∆Movrp1* mutant at least partially was caused by the defects in the normal regulation of extracellular peroxidase and laccase activities, and the secretion of cytoplasmic effectors such as Avr-Pia.

Autophagy plays an important role in nutrient recycling, cellular degradation, cell death, and cellular differentiation in the fungal life cycle^60^. In fungi, the autophagy pathway is not only regulated by a family of *ATG* genes such as *ATG4, ATG8*, and *ATG5*, but also by other genes such as *SCH9, ATG15, VPS34, ATG6*, and *ATG14*^61^,^62^,^63^,^64^. Thus, it is likely that autophagy may be impacted by other cellular signaling events. In *M. oryzae*, induction of autophagy during infection-related development is regulated in a manner depend on the Pmk1 MAP kinase pathway^65^. Mon1 proteins are involved in autophagy and are required for vacuolar assembly, conidiogenesis and pathogenicity in *Fusarium graminearum* and *M. oryzae*^66^,^67^. In this study, we showed that deletion of MoVrp1 affects the autophagy process. Since previous studies showed that infection-associated autophagy is required for the development of *M. oryzae*^60^,^68^, blocking autophagy may also affect fungal pathogenicity.

In summary, we identified MoVrp1, a putative verprolin protein, which is required for endocytosis, conidiation and pathogenicity in *M. oryzae*. In addition, we found that MoVrp1 plays a role in the activities of extracellular peroxidases and laccases, which reduce the ability to detoxify ROS and attenuate pathogenicity. Moreover, MoVrp1 plays a role in the secretion of effector proteins and autophagy and is required for full virulence in *M. oryzae*. Given the important roles of the MoVrp1 in the aspects of physiology and pathogenicity of *M. oryzae*, its interacting proteins and its expression regulation model should be emphasized in the further studies.

## Methods

### Fungal strains and culture conditions

*M. oryzae* wildtype Guy11 and all mutant strains were cultured in an incubator at 28 °C. The media plates of complete medium (CM), oatmeal medium (OMA), V8 juice agar medium (V8) and straw decoction and corn medium (SDC) were also used to culture fungal strains^69^. Liquid CM was used to culture and harvest the fungal mycelia for biomass analysis, genomic DNA and RNA extraction and protoplasts preparation as described^70^.

### Targeted gene deletion and complementation

The *MoVRP1* gene replacement construct was generated by a ligation-PCR approach following the method as described^71^. The upstream and downstream flanking sequences of *MoVRP1* were amplified with primers GKO005/GKO006 and GKO007/GKO008 (Table S1). Primers GKO006 and GKO007 contained the *Hind* III and *Spe*I sites, respectively. The resulting PCR products were digested with *Hind* III and *Spe*I and ligated with the hygromycin phosphotransferase (*HPH*) gene released from pCX62 by *Hind* III and *Spe*I digestion. After ligation, a 3.5-kb gene replacement fragment was amplified with primer set GKO005/GKO008 and purified and transformed into the protoplasts of *M. oryzae* wildtype Guy11. All amplified fragments were verified by sequencing.

To generate the MoVrp1-GFP fusion constructs, a 2.9-kb fragment with the *MoVRP1* open reading frame and its native promoter region was amplified and co-transformed with *Xho*I digested pYF11 into *S. cerevisiae* strain XK125^72^. The final plasmids pYF11-11243com was sequenced to contain the inframe MoVrp1-GFP fusion constructs and transformed into the *∆Movrp1* mutant according to the previous method^73^. To evaluate the effect of the disruption of *MoVRP1* on the secretion of effector Avr-Pia, the vector of *Avr-Pia:GFP* was constructed with the method as described^30^, and transformed into the *∆Movrp1* mutant and wildtype to generate the *AVR-Pia* expressed *∆Movrp1*/*AVR-Pia* and Guy11/*AVR-Pia* stains, respectively.

### Nucleic acid manipulation and Southern blot analysis

The standard Southern blot protocol was used as described^74^. Probe labeling, hybridization and detection were preformed according to the manual of DIG High Prime DNA Labeling and Detection Starter Kit (Roche Applied Science, Penzberg, Germany). Total RNA was isolated from fresh mycelia using the RNA extraction kit (Invitrogen, Carlsbad, CA, USA) and semi-quantitative RT-PCR was carried out to confirm the deletion and reintroduction of the *MoVRP1* gene were performed as described^40^.

### Vegetative growth, conidiation, osmotic sensitivity and surface hydrophobicity assays

Mycelial blocks of Guy11, *∆Movrp1* mutant and complemented strain (5 mm × 5 mm in size) were inoculated onto CM, V8, OMA and SDC media, respectively. The diameter of fungal colonies was measured after incubation for 7 days. Fungal biomass in liquid CM was examined as described^75^. The conidiophores and conidia of the wildtype Guy11, *∆Movrp1* mutant and complemented strain were induced on the SDC plates according to the previous method^41^. For stress assays, mycelial blocks (5 mm × 5 mm) were inoculated onto the CM agar plates with NaCl (0.7 M), KCl (0.6 M) and sorbitol (1 M and 2 M), SDS (0.005%, 0.01% and 0.02%), CFW (50 and 100 μg/mL) and CR (200, 400 and 600 μg/mL), or at the temperature of 20, 25, 28, 30 and 33 °C, respectively, and cultured in the dark for 7 days. Detergent solutions containing 0.02% SDS and 5 mM EDTA were used to determine the surface hydrophobicity. Detergent solution droplets were inoculated to the surface of 7-day old hyphae for 5 min. All experiments were repeated three times, with three replicates.

### Plant infection assays

For pathogenicity assays, mycelial mats of the wildtype Guy11, *∆Movrp1* mutants and complemented strain were cultured on liquid CM at 150 rpm for 2 days at 28 °C, and then inoculated on wounded and unwounded leaves of the susceptible rice variety CO39 and barley leaves as described^7^. Root infection assays were performed as described^76^. The experiments were repeated three times.

### Extracellular enzyme activities assay

Laccase activities of the wildtype Guy11, *∆Movrp1* mutants and complemented strain on CM medium plates supplemented with ABTs (Sigma) were examined as described^34^. In order to detect the peroxidase activities, a 5 × 5 mm hyphal plug was inoculated on CM medium plate containing 200 mg/ml CR and placed in an incubator at 28 °C for 7 days, then the CR degradation halo was observed. Peroxidase and laccase activities in culture filtrates were carried out as described^77^.

### qRT-PCR analysis

For evaluating the effect of deletion of *MoVRP1* on the expression levels of genes involved in pigmentation biosynthesis, conidiation, chitin synthesis, surface hydrophobicity, laccase and peroxidase, mycelial blocks of Guy11, *∆Movrp1* mutant were cultured on liquid CM at 150 rpm for 2 days at 28 °C, then the Total RNA was extracted as above. In order to exam the expression level of *AVR-Pia* in Guy11/*AVR-Pia* and *∆Movrp1*/*AVR-Pia*, mycelial mats of these strains were inoculated on the detached barley leaves. The mycelial mats were used to extract the total RNA for transcript analysis of *AVR-Pia* at 24, 48 hours post-inoculation (hpi), respectively. Total RNA were reverse transcribed into first-strand cDNA following the manual of PrimeScript™ II 1st Strand cDNA Synthesis Kit (TAKARA, Dalian, China) and used as the templates of qRT-PCR. qRT-PCR reactions were performed according to previously established procedures and *Actin* was used as internal control^40^. The experiment was carried out three times and each qRT-PCR had three replicates. Primer pairs used in this section were listed in Table S1.

### Light microscopy, confocal microscopy and transmission electron microscopy assays

The chitin deposited in the cell wall, hyphal apices and septa were observed by CFW (Sigma, St. Louis, MO, USA) staining as described^78^. FM4-64 staining was conducted following the procedures described previously^28^. Photographs were taken under a confocal laser scanning microscopy and the Leica DMR microscope (Leica Microsystems, Wetzlar, Germany). For autophagy assay, the wildtype Guy11 and ∆*Movrp1* mutant were cultured in liquid CM medium at 150 rpm for 10 h at 28 °C, and then shifted to liquid MM-N medium with 2 mM PMSF for 4 h. Transmission electron microscopy assay was carried out as described^7^.

## Additional Information

**How to cite this article**: Huang, L. *et al*. MoVrp1, a putative verprolin protein, is required for asexual development and infection in the rice blast fungus *Magnaporthe oryzae. Sci. Rep.* **7**, 41148; doi: 10.1038/srep41148 (2017).

**Publisher's note:** Springer Nature remains neutral with regard to jurisdictional claims in published maps and institutional affiliations.

## Supplementary Material

Supplementary Information

## Figures and Tables

**Table 1:** Inhibition rate of hyphal growth of *∆Movrp1* mutants exposed to the salt, osmotic and temperature stress (%).

	0.7 M NaCl	0.6 M KCl	2 M Sorbitol	20 °C	25 °C	30 °C	33 °C
Guy11	37.3 ± 1.5	34.0 ± 1.0	62.0 ± 2.4	56.3 ± 2.1	28.3 ± 3.8	39.0 ± 4.4	77.0 ± 1.7
*∆Movrp1#54*	44.7 ± 0.6*	40.0 ± 1.7*	65.0 ± 1.6*	25.7 ± 1.5*	2.3 ± 2.9*	2.3 ± 4.7*	72.2 ± 2.3*
*∆Movrp1#105*	45.3 ± 1.2*	41.7 ± 2.3*	66.5 ± 1.4*	29.3 ± 1.5*	2.7 ± 3.2*	2.5 ± 2.7*	68.3 ± 0.6*
*∆Movrp1/VRP1*	39.0 ± 1.0	32.0 ± 1.7	61.0 ± 2.4	56.6 ± 1.0	26.5 ± 0.9	38.9 ± 1.4	74.6 ± 1.9

±SD was calculated from data on three repeated experiments, and *t* test analysis was shown with ^*^p < 0.01 *versus* the wild type Guy11.

**Table 2:** Inhibition rate of hyphal growth of *∆Movrp1* mutants exposed to the cell wall stressors (%).

	0.005% SDS	0.01% SDS	0.02% SDS	50 μg/ml CFW	100 μg/ml CFW	200 μg/ml CR	400 μg/ml CR	600 μg/ml CR
Guy11	10.0 ± 1.0	37.0 ± 2.7	64.7 ± 1.2	51.1 ± 4.6	60.8 ± 3.2	18.0 ± 4.0	25.0 ± 1.7	30.0 ± 1.0
*∆Movrp1#54*	5.3 ± 1.5^*^	22.0 ± 2.7^*^	61.7 ± 2.5^*^	22.5 ± 1.2^*^	28.8 ± 1.7^*^	12.0 ± 0.2^*^	16.7 ± 1.5^*^	23.3 ± 2.5^*^
*∆Movrp1#105*	6.0 ± 1.7^*^	26.3 ± 1.5^*^	62.3 ± 1.4^*^	20.5 ± 2.2^*^	28.4 ± 1.6^*^	10.0 ± 1.7^*^	16.3 ± 1.2^*^	23.7 ± 2.9^*^
*∆Movrp1/VRP1*	16.7 ± 5.0	39.0 ± 1.0	66.7 ± 0.6	46.9 ± 2.4	62.6 ± 1.3	21.3 ± 0.6	29.0 ± 3.6	30.3 ± 1.5

±SD was calculated from data on three repeated experiments, and *t* test analysis was shown with *p < 0.01 *versus* the wild type Guy11.
