# Supplementary material for: MoVrp1, a putative verprolin protein, is required for asexual development and infection in the rice blast fungus Magnaporthe oryzae
Source: Sci Rep. 2017 Jan 24;7:41148. doi: 10.1038/srep41148 (PMC5259722; doi:10.1038/srep41148)
Supplement: Supplementary Information [file srep41148-s1.pdf]

MoVrp1, a putative verprolin protein, is required for asexual development and infection in the rice blast fungus *Magnaporthe oryzae*

Lin Huang[1,2], Shengpei Zhang[1], Ziyi Yin[1], Muxing Liu[1], Bing Li, Haifeng Zhang[1], Xiaobo Zheng[1], Ping Wang[3], and Zhengguang Zhang[1*]

[1]Department of Plant Pathology, College of Plant Protection, Nanjing Agricultural University, and Key Laboratory of Integrated Management of Crop Diseases and Pests, Ministry of Education, Nanjing 210095, China.

[2]Collegeof Forestry and Co-Innovation Center for Sustainable Forestry in Southern China, Nanjing Forestry University, Nanjing, Jiangsu 210037, China.

[3]Department of Pediatrics, Louisiana State University Health Sciences Center, New Orleans, Louisiana 70118, USA.

*Corresponding author: Zhengguang Zhang

E-mail: zhgzhang@njau.edu.cn

Tel: 86-25-84396972

Fax: 86-25-84396436

SUPPORTING INFORMATION

Figure S1. Phylogenetic analysis of MoVrp1 and its homologs from different organisms and targeted gene deletion and complementation. A, Phylogenetic tree of Vrp1 proteins was constructed based on alignment of the full sequences of Vrp1 from different fungi species: *Sclerotinia sclerotiorum* (XP_001592438), *Botrytis cinerea* (EMR89025), *Marssonina brunnea* (XP_007293786), *Magnaporthe oryzae* (XP_003714188), *Neurospora crassa* (XP_963859), *Nectria haematococca* (XP_003054239), *Fusarium oxysporum* (EWY94055), *Villosiclava virens* (KDB14436), *Beauveria bassiana* (EJP70600), *Cordyceps militaris* (XP_006670718), *Saccharomyces cerevisiae* (NP_013441), *Candida albicans* (EEQ47319). Phylogenetic trees of MoVrp1 homologues from several other species were drawn by the divergence distance method using the CLUSTAL_W program and the calculated phylogenetic tree was viewed using the Mega6.0 Beta program. UPGMA tree with 1000 bootstrap replicates of phylogenetic relationships. **B**, Semi-quantitative RT-PCR analysis the expression of *Movrp1* and *Actin* in the Wildtype Guy11, Δ*Movrp1* mutants and complemented strain. Data comprise three independent experiments with triple replications that yielded similar results. **C**, Southern blot analysis the Δ*Movrp1* mutants. Probe A, the hybridization probe generated from *HPH* gene. Probe B, the hybridization probe generated from *Movrp1*.

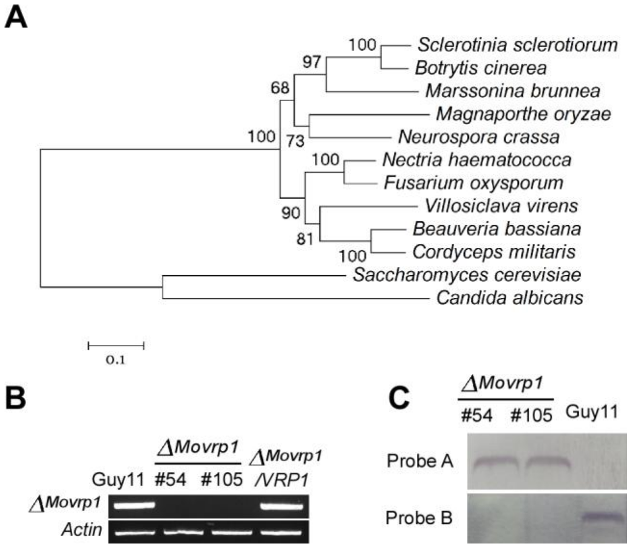

Figure S2. MoVrp1 is required for pathogenicity on detached barley leaves. A and **B**, Detached barley leaves, unwounded and wounded by abrasion, were inoculated with mycelial plugs of the wildtype Guy11, Δ*Movrp1*#54, Δ*Movrp1*#105 and complemented strain. Photographs were taken 5 days after inoculation. These experiments were performed three times with similar results.

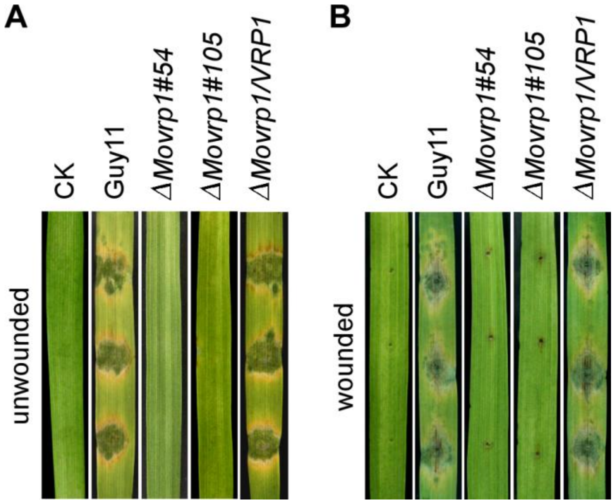

Table S1 Primers used in this study

Primer name	Primer Sequences (5'-3')	Remark
GKO005	CCTGCGTGTTTGTCCTGTCT	
GKO006	GGAAGCTTCCGGTCAAGCCGAGTCAGAA	*Movrp1* deletion vector
GKO007	GGACTAGTGTGGATTGGGTAGAGACAT	construction
GKO008	CCACCCCATCCACAACAATG	
11243COM_F_113	ACTCACTATAGGGCGAATTGGGTACTCAAATTGGTTGCCCGTAGCG TGGAAGCCT	complemented vector
11243COM_R	CACCACCCCGGTGAACAGCTCCTCGCCCTTGCTCACCCCCAGTGA TCTTAGATCCAATG	construction of *Movrp1*
STHN11243F	ACCACCGTCAATGTCTGCTC	amplification of *Movrp1*
STHN11243R	GTGGCTTCGGAAACTTGTCTT	probe for southern blot
FL1111	GGAGGTCAACACATCAATG	amplification of *HPH* probe
FL1112	CTCTATTCCTTTGCCCTCG	for southern blot
RT11243F	TCCGAGCAGCATCAAGTCC	semi-quantitative RT-PCR
RT11243R	CCACCATTGGCACCATCAC	of *Movrp1*
ACTIN_F	CCATGTACCCTGGTCTTTCG	semi-quantitative RT-PCR
ACTIN_R	TTCGAGATCCACATCTGCTG	and qRT-PCR primer of *MoACTIN*
MPG1_QF	GAGAAGGTCGTCTCTTGCTG	qRT-PCR primer of
MPG1_QR	TGTCCGAGCAGAAGTTGTTG	*MoMPG1*
MHP1_QF	CATCATCGCCACCATCTTC	qRT-PCR primer of
MHP_QR	CTGGCCACAGTCGAGGTT	*MoMHP1*
MGG10105-QF	CGGCAGCGGAGACTATGA	qRT-PCR primer of
MGG10105-QR	CGCAAATGTCGGTGAAGC	*MGG_10105*
MGG09134-QF	GCAGCGGAGCCTACAACAA	qRT-PCR primer of
MGG09134-QR	TCCAAGAACAGGGAGCAGACA	*MGG_09134*
FL4739	CCCTCAGCCCACATACAACT	qRT-PCR primer of
FL4740	AGCCTTCGCTCGATACTGAA	*MoCOS1*
FL9560	ACCGATTCTGACGAATCCAG	qRT-PCR primer of
FL9561	CTGGAACTGCTGTCCTCCTC	*MoCOM1*
FL9562	GCAAGAAGTGCGTTCAAACA	qRT-PCR primer of
FL9563	TCTCCACTGCTGCCACTATG	*MoCON7*
FL12980	CACAAGGCCAACCTCAA	qRT-PCR primer of
FL12981	TCTCCATCTCCTCGAGAC	*MoCON6*
FL12982	GATTCATCCGCAGCAAA	qRT-PCR primer of
FL12983	CACATCTTGCCAAACAGG	*MoCON8*
FL12984	CGATAATTGCTCCCACACCT	qRT-PCR primer of
FL12985	GAAGGAGTCGGTGGTGACAT	*MoHOX2*
FL4708	GCAATGTCGGTCCCAACTAC	qRT-PCR primer of
FL4709	ATCTCAAAGGCGATGACACC	*MoALB1*
FL4710	CGACTCCAAGGACTGGGATA	qRT-PCR primer of

FL4711	GTCCTCGGACACCTTCTCC	*MoRSY1*
FL4712	ACGCCGTCTACTCAGGATCA	qRT-PCR primer of
FL4713	TCTCGCCGTTTGGAATGTAT	*MoBUF1*
FL4929	TGCTGCTCATGTCCACCTAC	qRT-PCR primer of *CTS1*
FL4930	TCCTCTTGAGGCTTGTCGAT	
FL4931	CTTGTTGCAAAGCGAGATGA	qRT-PCR Primer of *CTS2*
FL4932	CCTGGAGAAGCTGGTAGACG	
FL4933	CGACCAGCTTCAACTTCACA	qRT-PCR primer of *CTS3*
FL4934	GGAGTCTGAGCTTCGTTTGG	
FL4935	AAACTCGAGGGACATGTTGG	qRT-PCR primer of *CTS4*
FL4936	CCTCCTGAACGCAGAGAAAC	
FL4937	TCCTGATGTCGTTCTTGCAG	qRT-PCR primer of *CTS5*
FL4938	GATCTCAGGGTCCTTCACCA	
FL4939	TATGCGCTACGATGACAAGC	qRT-PCR primer of *CTS6*
FL4940	CGAGTAAACCTTGCCCATGT	
FL4941	CACCTGCGTTTACCTTGGAT	qRT-PCR primer of *CTS7*
FL4942	TACCCCACGAGAAGTTGTCC	
FL4368	CGTCCACTGCCACATCGC	qRT-PCR primer of
FL4369	AGTCGTCCTGGTGGAAAGG	*MGG_11608*
FL4370	TGTTCCACTGCCACATCG	qRT-PCR primer of
FL4371	CTCAGACCAGAGTCGTGCTG	*MGG_13464*
FL16892	TCGAGGCTGATGGGATTCT	qRT-PCR primer of
FL16893	ATGCTGCCGTAGGGATTATG	*MGG_00551*
FL16890	ACCTGATAGCGGGATTTTG	qRT-PCR primer of
FL16891	GGCCGACGTAAGTGATGTT	*MGG_02156*
FL16894	TGGCCCAATGGTCTTCTAT	qRT-PCR primer of
Fl16895	TGCCGTTGATCAGGTTGTT	*MGG_02876*
FL16902	CCTACCCCGACAACATCAT	qRT-PCR primer of
FL16903	GCCGTCCATCTCCACTATC	*MGG_07771*
FL16908	AAGTGGAAGGCGGAACAGTA	qRT-PCR Primer of
FL16909	GCCAGTCGGAGAGGATGAT	*MGG_08523*
FL4801	ACCAACAACCTCACCAGGAG	qRT-PCR primer of
FL4802	GCTGGACCGTTGATGACGAT	*MGG_09103*
FL16912	TGATCAACGGTACCAATGTCTA	qRT-PCR primer of
FL16913	CCTATGCCGATGCTTACCA	*MGG_09139*
FL16918	GGCCAGTACCAACAAGACAA	qRT-PCR primer of
FL16919	AAGGGCTGCAGGGTAAACT	*MGG_14307*
FL16896	CTCGCTTGACAACCACACC	qRT-PCR primer of
FL16897	CCGAGGCTTGACCGTAGTAG	*MGG_05790*
FL16898	CGCTACGACGTGCTCATCT	qRT-PCR primer of
FL16899	GTCGCCAACAACATCGTAAG	*MGG_07220*
FL16900	CGCTACGACGTGCTCATCT	qRT-PCR primer of
FL16901	GTCGCCAACAACATCGTAAG	*MGG_07500*
FL13047	TTACAACGACCCCAACTTCAT	qRT-PCR primer of

FL13048	CCTTTCCCGCTGTCATTCT	*MGG_08046*
FL13043	CAGTACATCGCGGAGAAAAA	qRT-PCR primer of
FL13044	GACGGGGTTATTGAAGTTGAG	*MGG_08127*
FL13045	GTCAACAACGCTGCTCTCC	qRT-PCR primer of
FL13046	GGGGTCCGTGATGATGTAG	*MGG_13764*
Pia-F	TTTCGTAGGAACCCAATCTTCAAAATGCATTTTTCGACAATTTTC	*AVR-Pia::GFP* vector construction
Pia-R	CACCACCCCGGTGAACAGCTCCTCGCCCTTGCTCACGTAAGGCTCGGCAGCAAGCC	
Pia-Q-F	GCCAGCTAGATTTTGCGTCT	qRT-PCR primer of
Pia-Q-R	CTTGTTTGCCATTGGTGAGA	*AVR-Pia*
